# Lessons Learned from a Global, Multisite Early Detection Program in Primary Care Implementation Program

**DOI:** 10.1002/alz.090782

**Published:** 2025-01-09

**Authors:** Tim MacLeod

**Affiliations:** ^1^ Davos Alzheimer’s Collaborative, Wayne, PA USA

## Abstract

Davos Alzheimer’s Collaborative Healthcare System Preparedness (DAC‐SP) aims to catalyze global healthcare system transformation, providing patients with quicker access to life‐changing innovations and therapies. Utilizing implementation science, the DAC‐SP Early Detection flagship program launched in 2021, engaging seven healthcare systems across six countries (Brazil, Jamaica, Japan, Mexico, Scotland, and the United States). The program's primary aim was to increase the rate of early detection of cognitive impairment by integrating commercially available digital cognitive assessments (DCAs) into primary care and other non‐specialty care settings. Patients with suspected cognitive decline based on the DCA were then offered a blood‐based biomarker (BBM) to determine the likelihood of amyloid presence in addition to customary follow‐up testing.

Over the past two years, health system leaders from the early detection program were convened into a Community of Practice (CoP) that met monthly to discuss operational and clinical challenges, collaborate on solutions, and exchange learnings. As part of the CoP meetings, site leaders identified common implementation challenges for DCAs and BBMs in real‐world primary care settings. These challenges span various levels of health systems, including policy (e.g., reimbursement and procurement mechanisms, lack of guidance and consensus on workflow), the outer setting (e.g., availability/capacity of brain health expertise, lab infrastructure, fit with administrative systems), the inner setting (e.g., training and equipment required for blood draws, shipping logistics, administrative support) and understanding clinician knowledge, attitudes and behavior (e.g., level of comfort or experience with cognitive assessments, interpretation and disclosure of BBM results, access to training, task shifting resources, support from peers). Education about BBM specifications, use cases, and integration into clinical routines was essential in understanding the adoptability and scalability of BBMs in primary care settings.

